# Intranasal delivery of blackberry-loaded Chitosan nanoparticles for antipsychotic potential in Ketamine-induced schizophrenia in rats

**DOI:** 10.1038/s41598-025-00918-2

**Published:** 2025-05-14

**Authors:** Mina Y. George, Nermeen Farag, Esther T. Menze, Reham S. Elezaby, Nayera A. Salem, Shereen K. Elrahmany, Nahed Adel, Rana M. ElKhatib, Menna Galal, Hams Assem, Radwa Elkhouly, Mariam Alkess Wesa, Noha Hesham, Nouran Hossam, Iriny M. Ayoub

**Affiliations:** 1 Department of Pharmacology & Toxicology, Faculty of Pharmacy, Ain Shams University, Abbassiya, Cairo, 11566 Egypt; 2 Department of Pharmacognosy, Faculty of Pharmacy, Cairo University, Cairo, Egypt; 3 Department of Pharmaceutics, Faculty of Pharmacy, Ain Shams University, Abbassiya, Cairo, 11566 Egypt; 4 Drug Design Program Graduates, Faculty of Pharmacy, Ain Shams University, Abbassiya, Cairo, 11566 Egypt; 5 Department of Pharmacognosy, Faculty of Pharmacy, Ain Shams University, Abbassiya, Cairo, 11566 Egypt

**Keywords:** *Rubus fruticosus*, Anthocyanins, UPLC-ESI-MS/MS, Chitosan, Schizophrenia, Clozapine, Neurological disorders, Blood-brain barrier

## Abstract

Schizophrenia is a neuropsychiatric disorder with limited treatment options that have unwanted side effects. Clozapine, an atypical antipsychotic, has been used for resistant schizophrenia. This study investigated the effect of anthocyanin-rich extract from *Rubus fruticosus* (RFE) loaded into chitosan nanoparticles against ketamine-induced schizophrenia in rats. The extract’s phenolic and flavonoid content was measured using UPLC-ESI-MS/MS, revealing 9.42 ± 0.5 mg of gallic acid equivalent and 2.54 ± 0.02 mg of quercetin equivalent per g of extract, with 19 identified compounds, predominantly anthocyanin glycosides. Chitosan nanoparticles were prepared with chitosan and sodium tripolyphosphate at a ratio of 3:1 with particle size 194.49 ± 5.69 nm and encapsulation efficiency of 64.6 ± 1.12%. Rats were treated with ketamine to induce schizophrenia-like symptoms, and various groups received different treatments, including control, ketamine, ketamine + clozapine, ketamine + RFE, and a combination of clozapine/RFE. Clozapine and RFE treatments were initiated from day 8 to day 14. RFE treatment ameliorated positive, negative, and cognitive symptoms of schizophrenia while mitigating clozapine-induced side effects such as weight gain, hyperglycemia, hyperlipidemia, agranulocytosis, and liver dysfunction. RFE corrected oxidative stress as evidenced by its effect on catalase activity and reduced glutathione level. Additionally, RFE hindered neuroinflammation induced by ketamine and reduced the levels of Tumor Necrosis Factor-α. RFE also increased BDNF levels. This study demonstrates that RFE-loaded chitosan nanoparticles exhibit potent antipsychotic properties and enhance clozapine’s therapeutic efficacy while minimizing its adverse effects. This could shed light on the integration of the use of natural products and advanced nano-based formulations to manage schizophrenia patients worldwide.

**Supplementary Information:**

The online version contains supplementary material available at 10.1038/s41598-025-00918-2.

## Introduction

Schizophrenia is a multifactorial disabling disorder affecting people worldwide. The symptoms triad, positive, negative, and cognitive symptoms, affects people′s life socially and mentally^1^. The pathogenesis of schizophrenia may be related, but not limited to, glutamate excitotoxicity, oxidative stress, cytokines production, neurotrophic factors, and cholinergic abnormalities^2,3^. Schizophrenia was previously reported to be associated with enhanced glutamate levels, which in turn could induce hyperdopaminergic functioning^2^. In addition, free radicals formation in schizophrenia reduces the body’s antioxidant capacities and enhances lipid peroxidation^4^. Moreover, the inflammatory response resulting from oxidative stress leads to the release of inflammatory cytokines, contributing to the pathogenesis of schizophrenia^5^. Neurotrophins, as BDNF, which play an important role in cell survival and development were found to be affected in schizophrenia^6^. Besides, cholinergic abnormalities may lead to cognitive symptoms seen in schizophrenia^7^.

NMDA receptor antagonism was among the models used to study schizophrenia preclinically. Ketamine sub-anesthetic repeated administration in animals mimics positive, negative, and cognitive symptoms of schizophrenia^8^. This induces the development of behavioral changes associated with schizophrenia. Clozapine (CLZ), the first atypical antipsychotic drug with dopamine (D2) receptors blocking activity, is effective but limited by severe side effects such as weight gain, metabolic disturbances, and agranulocytosis^9^. On the other side, CLZ was reported to induce metabolic abnormalities including weight gain, hyperlipidemia, hyperglycemia, and leucopenia owing to its effect on histamine and serotonin receptors^10^. Moreover, it was reported that CLZ-induced hepatic impairment in schizophrenia patients^11^. In addition to these side effects, CLZ was not effective in reversing oxidative damage in schizophrenia models in rodents^12^. Therefore, CLZ was used for treatment-resistant schizophrenia.

*Rubus fruticosus* (blackberry) is native to Europe and has spread to the Americas, Australia, and Asia^13^. Its fruit is rich in polyphenols, including phenolic acids, tannins, and anthocyanins, and contains carbohydrates, vitamins (C, K, E, A), minerals (potassium, iron, calcium), and dietary fiber^14,15^.

Various parts of the plant have been claimed to be useful in ailments like diabetes, diarrhea, and epilepsy and as a wound healing agent, antimicrobial and as renal tonic^16^. In vitro studies have revealed blackberries possess potent antioxidant, anti-carcinogenic, antiproliferative, and anti-inflammatory activities^17^. Interestingly, the effect of chronic consumption of blackberry extract on high-fat induced obesity in rats and its correlation with metabolic and brain changes showed improved glucose metabolism, decreased levels of brain-derived neurotrophic factor (BDNF), and it also affected the dopaminergic system by increasing dopamine turnover in the striatum^18^. Blackberry extract has also prevented memory deficits and neurochemical alterations, mainly by affecting the oxidative stress levels and acetylcholinesterase activity in the cerebral cortex, hippocampus, and cerebellum of a rat model with amnesia^19^. Similarly, blackberry extract exhibited neuroprotective, antioxidant and anti-inflammatory effects in the ketamine-induced rat model of mania^20^.

Chitosan is an amino polysaccharide obtained by alkaline deacetylation of chitin^21,22^. It consists of D-glucosamine and N-acetyl-D-glucosamine, a straight homopolymer built by *β*-*(*1,4*)*-linked N-acetyl-glucosamine units^23^. Chitosan is typically insoluble in water due to the strong intermolecular and intramolecular hydrogen-bonding interactions between its chains. However, chitosan solutions can be obtained in acidic aqueous media that protonate chitosan amino groups, rendering the polymer positively charged and thereby overcoming associative forces between chains. In the pharmaceutical field, chitosan has many advantages: it is a biodegradable, biocompatible, and mucoadhesive nontoxic cationic polymer^22,24^. The positive charge on the chitosan chains is responsible for the mucoadhesion property where a strong electrostatic interaction occurs with the negative components of the mucous membrane such as sialic acid. Meanwhile, hydrogen bonds and hydrophobic interaction share in the interaction with mucous membrane^25,26^. Chitosan also acts as a permeation enhancer by opening the tight junctions and enhancing both the paracellular and transcellular transport^27,28^.

Owing to the combined properties of mucoadhesion, permeation enhancement, biocompatibility, and biodegradability, chitosan nanoparticles have been considered as very promising drug delivery systems during the last decades. In addition to the aforementioned advantages of chitosan nanoparticles, chitosan possesses a powerful antioxidant activity due to its free radical scavenging ability^29,30^ which is very beneficial in the treatment of psychosis. Moreover, chitosan nanoparticles are excellent for the encapsulation of hydrophilic drugs, and they are prepared in an acidic medium, which is optimum for anthocyanin stability.

Intranasal administration has shown promise for both systemic and brain delivery^31,32^. The septum, which has a total surface area of 150 cm^2^ for the nasal epithelium and a total volume of 15 ml, divides the nose into two nasal cavities. The nasal vestibule, respiratory, and olfactory regions are the three separate sections that make up nasal cavities. Systemic drug absorption occurs either transcellularly or paracellularly through the respiratory area. Meanwhile, the olfactory region was the focus of numerous recent projects where direct-to-brain delivery occurs. The advantages of the intranasal include (i) non-invasive character, with no dangers of transmission of infection or sickness, (ii) easy self-administration, (iii) large surface area of absorption, (iv) rapid effect, and (v) a direct route to the brain that avoids the blood-brain barrier and the first pass effect. However, its drawbacks include (i) a small administration volume (25–200 µl), which limits the use of high-dose medications; (ii) individual variability; (iii) a low nasal cavity pH (5.5–6.5 in adults); (iv) mucociliary clearance; and (v) enzymatic degradation by nasal cytochrome P-450/peptidases/proteases (pseudo-first pass effect).

In the current study, an anthocyanin-rich extract of *Rubus fruticosus* (RFE) was prepared and was fully characterized in terms of total phenolic and total flavonoid content. Furthermore, metabolite profiling was performed using UPLC-ESI-MS/MS. Chitosan nanoparticles were chosen as smart carriers for intranasal brain delivery of RFE. The primary target of the present research was to demonstrate the potential therapeutic effects of anthocyanin-rich extract of *R. fruticosus* (RFE) and/or CLZ/RFE combination against ketamine-induced schizophrenia in rats, as well as the possible underlying mechanisms. The secondary target was to investigate the effect of RFE on the main CLZ adverse effects.

## Materials and methods

### Plant material

Blackberry fresh fruits were purchased from Gourmet^®^ Egypt. Fruits were kindly authenticated by Mrs. Therease Labib, Consultant of Plant Taxonomy at the Ministry of Agriculture and Orman Botanical Garden, Giza, Egypt. A voucher specimen (PHG-P-RF-497) was deposited at the Herbarium of Pharmacognosy Department, Faculty of Pharmacy, Ain Shams University, Cairo, Egypt.

### Animals

Male Wistar rats (200–220 g) were purchased from Nile Co. for Pharmaceutical and Chemical Industries, Cairo, Egypt. They were left for acclimatization for 2 weeks before experimentation. Rats were kept in a suitable atmosphere (25 °C) with alternatively 12 h light and dark cycles and given food and water *ad libitum.*

### Drugs and chemicals

Chitosan medium molecular weight (Mwt = 100–300 KD) was purchased from Acros organics (USA), and sodium tripolyphosphate (TPP) was purchased from Alfa Aesar (USA). Ketamine was purchased as ketamine hydrochloride (Rotexmedica Company, Ttittau, Germany). CLZ was purchased as Clozapex^®^ from Multi-Apex for pharmaceutical industries S.A.E. (Egypt) and Sodium Carboxymethyl cellulose (Na CMC) was purchased from Sigma Aldrich (St. Louis, MO, USA). Glacial acetic acid and hydrochloric acid were purchased from Piochem (Egypt). All other chemicals and buffers were of the highest purity grade commercially available.

### Preparation of the plant extract

Plant extraction was performed following the method previously described by Wang et al.^33^. Reports indicated that the ideal composition for water-ethanol mixtures ranged from approximately 40–70% ethanol. To improve the extraction of anthocyanins, the solvent can be acidified, typically by 1%, using acids such as HCl or acetic acid. Ethanol-water mixtures were among the most commonly used solvents due to their cost-effectiveness, the renewable nature of ethanol (derived from sugarcane), and its classification as a GRAS (Generally Recognized as Safe) solvent, supporting a green chemistry approach^34^. Blackberry fruits were homogenized using a hand blender. Blackberry homogenate (110 g) was percolated three times in 500 mL of 70% acidified ethanol (Ethanol: water: HCl, 70:30:1) at a temperature of 20–25 °C for 48 h. The extract was filtered and centrifuged for 10 min at 4000x. The supernatant was concentrated under reduced pressure using a rotary evaporator (BUCHI, Switzerland) at a temperature not exceeding 45 °C. Subsequently, the extract was lyophilized using an Alpha 1–4 LSC Christ freeze dryer (Martin Christ Gefriertrocknungsanlagen GmbH, Osterode, Germany) to yield 10.85 g, which was kept at 4 °C in a tight container for further analysis.

### Quantitation of total phenolics content

Spectrophotometry was adopted for the quantification of total phenolics using the Folin Ciocalteu colorimetric method^35^. Gallic acid was employed as a reference standard. The assay was carried out in triplicate. The total phenolics content was expressed as mg gallic acid equivalent (GAE)/g dried fruit extract (DW).

### Quantitation of total flavonoids content

A spectrophotometric method was conducted in triplicate using an aluminum chloride reagent^36^. Quercetin was employed as a reference standard. The total flavonoid content was computed as a mg quercetin equivalent (QE)/g dried fruit extract.

###  Characterization of *Rubus fruticosus* fruit extract using UPLC-ESI-MS/MS analysis

Metabolite profiling of *Rubus fruticosus* fruit extract (RFE) was performed using UPLC-ESI-MS/MS analysis in both positive and negative ionization modes as previously reported^37^. Briefly, *R. fruticosus* fruit extract was dissolved in methanol (HPLC grade) at a concentration of 1 mg/mL, then filtered using a 0.2 μm membrane disc filter, and subsequently degassed by sonication prior to injection. Chromatographic separation was achieved by injection of 10 µL of the extract into UPLC equipped with a reversed-phase C-18 column (ACQUITY UPLC - BEH, 2.1 × 50 mm column; 1.7 μm particle size) coupled to an A XEVO TQD triple quadrupole mass spectrometer (Waters Corporation, Milford, MA01757 U.S.A). Gradient elution was applied at a flow rate of 0.2 mL/min. The mobile phase was composed of water acidified with 0.1% formic acid (eluent A) and methanol acidified with 0.1% formic acid (eluent B). Elution was carried out as follows: from 0 to 5 min, 10% B; from 5 to 15 min, 30% B; from 15 to 22 min, 70% B; from 22 to 25 min, 90% B and finally 25–29 min, 100% B. Mass analysis was set as follows: cone voltage 30 eV, capillary voltage 3 kV, source temperature 150 °C, desolvation temperature 440 °C, cone gas flow 50 L/h, and desolvation gas flow 900 L/h. Mass spectra were acquired in both positive and negative ionization modes (*m/z* 100–1000). Data were processed using Masslynx 4.1 software. Tentative identification was achieved by comparison of retention times (R_t_) and mass spectral data with those reported for *Rubus fruticosus* and online public databases.

###  UV scanning of *Rubus fruticosus* fruit extract in 2% acetic acid

*R. fruticosus* fruit extract dissolved in 2% acetic acid was scanned spectrophotometrically using a UV-visible spectrophotometer. The maximum wavelength (λ _max_) was determined.

###  Linear regression analysis of *Rubus fruticosus* fruit extract in 2% acetic acid

Serial dilutions of *R. fruticosus* fruit extract ranging from 40 to 200 µg/mL in 2% acetic acid were prepared and their absorbances were measured at the predetermined λ_max_ using 2% acetic acid as a blank. The measured absorbances were plotted against the corresponding concentrations and linear regression analysis calculations were done.

### Preparationand optimization of *Rubus fruticosus* fruit Extract/Chitosan nanoparticles (RF/CSNPs)

Chitosan nanoparticles (CS NPs) were prepared according to the ionic gelation method with optimization of different parameters such as CS and TPP concentrations, CS: TPP volume ratio, and pH to achieve optimal particle size and encapsulation efficiency^38,39^. A stock solution of CS (0.5%) in acetic acid (2% v/v, pH 3.5) was prepared under constant stirring at 300 rpm overnight, and a stock solution of TPP (1%w/v) was also prepared in distilled water (pH 9). NPs were obtained by the dropwise addition of TPP solution to CS solution with constant stirring at 600 rpm at room temperature for 30 min at different dilutions and volume ratios as shown in Table 1. Ten milligrams of *R. fruticosus* fruit extract was added to chitosan solution with constant stirring prior to the addition of TPP solution in all preparations. After optimization of the preparation conditions, the selected formula was further optimized by reducing the pH of the TPP solution to pH 7 and pH 2 using conc. HCl (Table 4).

**Table 1:** Composition of ***Rubus fruticosus fruit Extract***/Chitosan nanoparticles.

Formula code	Chitosan concentration (%w/v)	TPP concentration (%w/v)	CS: TPP (v/v)
F1	0.1%	0.1%	3:1
F2			6:1
F3	0.3% 0.1% 0.1% 0.1%	0.3% 0.1% 0.1% 0.1%	3:1
F4 F5 F6 F7			6:1 3:1 3:1 3:1

### Characterization of the prepared *Rubus fruticosus* fruit Extract/Chitosan nanoparticles (RF/CSNPs)

#### Particle size, polydispersity index and zeta potential

Particle size (P.S, the mean diameter), polydispersity index (PDI) and zeta (**ξ**) potential of all the prepared CSNPs were determined using a dynamic light scattering (DLS) particle size analyzer (Malvern zetasizer, Nano ZS, Malvern Instruments, UK) after appropriate dilution. All measurements were performed at a fixed angle of 173 º and a temperature of 25 ºC^40,41^.

#### Encapsulation efficiency

The CSNPs in the form of pellets were separated from the solution by ultracentrifugation at 14,000 rpm at 4 ºC for 30 min. The supernatant was carefully decanted and analyzed by UV spectrophotometer for Anthocyanins at the predetermined λ_max_. The %EE was calculated using the equation as given below^38^:

#### Experimental design

Seventy-two rats were randomly assigned to six groups (12 animals each) and treated for 14 days as follows: The first group (Control group) received the respective vehicles. The second group received *i.p*. ketamine at a dose of 25 mg/kg daily for 14 consecutive days^42^. The third group received *i.p.* ketamine at a dose of 25 mg/kg daily for 14 consecutive days and from day 8 to day 14, they received *i.p.* CLZ (5 mg/kg) one hour later after ketamine treatment. The fourth and fifth groups received *i.p.* ketamine at a dose of 25 mg/kg daily for 14 consecutive days. Then, from day 8 to day 14, they received one hour later daily intranasal RF/CSNPs (0.5 mg/kg) and intranasal RF/CSNPs (1 mg/kg), respectively. The sixth group received *i.p*. ketamine at a dose of 25 mg/kg daily for 14 consecutive days. In addition, from day 8 to day 14, rats were treated with a combination of *i.p.* CLZ (5 mg/kg) (one hour later) and intranasal RF/CSNPs (1 mg/kg).

Afterward, rats were subjected to behavioral assessments: locomotor activity and prepulse inhibition of acoustic startle reflex tests to assess positive symptoms, social interaction, splash tests to assess negative symptoms, and passive avoidance to assess cognitive symptoms. On day 14 of the experiment, rats were sacrificed by cervical dislocation, blood glucose was measured, blood samples were collected in heparinized tubes for further biochemical blood analysis and skulls were split on ice and salt mixture. Striata, hippocampi, and prefrontal cortices were dissected out and homogenized at 1:10 (*w/v*) in potassium phosphate buffer (pH 7.5) for further biochemical analyses.

### Behavioral tests

#### Locomotor activity

Locomotor activity was monitored by placing each rat in the recording chamber of Opto-Varimex-Mini Model B, Columbus Instruments, Columbus, OH, USA. Two hours after the last injection on day 14, rats were placed in the recording chamber for 2 min and then activity was determined for 5 min and expressed as counts per 5 min^43^.

#### Percentage prepulse inhibition (% PPI) of acoustic startle stimuli

The acoustic startle response of sensorimotor gating was determined using the Startle Responder X apparatus (Columbus, OH, USA). PPI response was then calculated where: % PPI = [(mean peak amplitudes on pulse alone sessions - mean peak amplitudes on prepulse/pulse sessions)/mean peak amplitudes on pulse alone sessions] ×100^44^.

#### Social interaction

On day 14, two rats from the same group (not subjected to each other before) were gently placed in a cage with dimensions 80 cm x 40 cm x 30 cm. Rats were left for 5 min to acclimatize, and then their behavior was monitored using a digital camera for 10 min. The active time of interaction was recorded (sec) for each pair of rats and the average interaction time per treatment group was calculated^45^.

#### Splash test

10% sucrose solution was sprayed on each rat body in a separated familiar cage. Grooming time for each rat was recorded over 10 min. Behavior was recorded using a digital camera^46^.

#### Step-through passive avoidance

Two-day passive avoidance test was conducted where a training session was held on day 13 and a testing session on day 14 using a Step-through passive avoidance apparatus (Ugo Basile, Italy). The apparatus consists of a Plexiglas box divided into light and dark compartments. On day 13, rats that failed to step through within a cut-off period of 90 s were excluded from the testing session. 24 h later, each rat was tested using the same procedure except for the foot shock delivered. The step-through latency to enter the dark compartment was measured indicating memory functioning^47^.

### Assessment of oxidative stress markers

Catalase (CAT) enzyme activity in the striata, hippocampi, and prefrontal cortices homogenates was assessed using kits purchased from Biodiagnostics, Giza, Egypt, according to Aebi^48^. Enzyme activities were expressed as unit/gm tissue. Reduced glutathione (GSH) was determined colorimetrically using kits provided by Biodiagnostics, Giza, Egypt in accordance with Beutler et al.^49^. The results were expressed as nmol GSH/gm tissue. Moreover, lipid peroxidation was determined by estimating the level of thiobarbituric acid reactive substances (TBARS) measured as malondialdehyde (MDA), according to the method of Satoh^50^. The results were expressed as nmol MDA/gm tissue using 1,1,3,3-tetraethoxypropane as standard.

### Assessment of BDNF and TNF-α

Both TNF-α and BDNF levels in striata, prefrontal cortices and hippocampi homogenates tissue were assessed using enzyme-linked immunosorbent assay kits for TNF-ɑ obtained from Biolegend, Inc. [(San Diego, USA); Catalog number 438204] and for BDNF obtained from Elab science [(USA); Catalog number E-EL-R1648]. A quantitative sandwich immunoassay technique was used for measuring each of these 2 parameters. Briefly, each kit had a microplate pre-coated with a monoclonal antibody against its legand. After adding the standards and samples, the TNF-α and BDNF antigens were sandwiched by the corresponding immobilized antibody and the biotinylated polyclonal antibody specific to them. The latter was recognized by a streptavidin–peroxidase conjugate. Afterward, washing removed any unbound proteins. Thereafter, a peroxidase enzyme substrate was added followed by the stop solution. Finally, the color intensity was measured at 450 nm using a microplate reader (ChroMate-4300, FL, USA). The intensity of the color was directly proportional to the marker concentration. The quantities of rat TNF-α and BDNF were expressed as pg/gm tissue.

### Body weight measurements

Initial and final body weights on day 1 and day 14 for all groups were recorded. Percentage change in body weights was determined as follows^51^:

% Change in body weight = $\:\frac{\text{F}\text{i}\text{n}\text{a}\text{l}\:\text{W}\text{e}\text{i}\text{g}\text{h}\text{t}\:-\:\:\text{I}\text{n}\text{i}\text{t}\text{i}\text{a}\text{l}\:\text{W}\text{e}\text{i}\text{g}\text{h}\text{t}}{\:\text{I}\text{n}\text{i}\text{t}\text{i}\text{a}\text{l}\:\text{W}\text{e}\text{i}\text{g}\text{h}\text{t}}$ × 100

### Hematological examination

Initial (day1) and final (day 14) blood glucose was measured using Accu-check glucose meter. The percentage change in blood glucose was calculated.

% Change in blood glucose = $\:\frac{\text{B}\text{l}\text{o}\text{o}\text{d}\:\text{g}\text{l}\text{u}\text{c}\text{o}\text{s}\text{e}\:\text{a}\text{t}\:\text{d}\text{a}\text{y}\:14\:-\:\:\text{B}\text{l}\text{o}\text{o}\text{d}\:\text{g}\text{l}\text{u}\text{c}\text{o}\text{s}\text{e}\:\text{a}\text{t}\:\text{d}\text{a}\text{y}\:1}{\:\text{B}\text{l}\text{o}\text{o}\text{d}\:\text{g}\text{l}\text{u}\text{c}\text{o}\text{s}\text{e}\:\text{a}\text{t}\:\text{d}\text{a}\text{y}\:1}$ × 100

Besides, cholesterol and triglycerides were determined using CHOP-PAP and GPO-PAP enzymatic colorimetric assays, respectively^52,53^. Results were expressed as gm/dl. Moreover, serum ALT and AST were determined in the serum of different treatment groups using Spectrum diagnostic kits purchased from the Egyptian company for biotechnology S.A.E., Cairo, Egypt (Catalog number #264002 and #260002, respectively). Results were expressed as U/L. In addition, whole blood samples from all groups were analyzed using an automated hematology analyzer (Sysmex kx-2 N, USA) to detect total leukocyte count to assess agranulocytosis.

### Statistical analysis

Data are presented as mean ± SD. Non-parametric data (Social interaction and Passive avoidance tests) were analyzed by Kruskal-Wallis test followed by Dunn’s as a post hoc test. For the rest of the data, multiple comparisons were performed using one-way ANOVA followed by Tukey as a post-hoc test. The 0.05 level of probability was used as the criterion for significance. All statistical analyses were performed using GraphPad Instat software version 3. Graphs were sketched using GraphPad Prism software version 9 (GraphPad Software, Inc., La Jolla, CA, USA).

## Results

### Assessment of total phenolics and total flavonoids content in *Rubus fruticosus* fruit extract (RFE)

The total phenolic content in RFE extract was 9.42 ± 0.5 mg gallic acid equivalent (GAE)/g of *R. fruticosus* fruit extract (DW). The total flavonoids content in RFE was 2.54 ± 0.02 mg quercetin equivalent (QE)/g *R. fruticosus* fruit extract (DW). The total phenolic content in *R. fruticosus* fruit extract calculated herein was consistent with previous studies. Calent et al. reported the total polyphenol concentration ranging from 8.23 to 14.98 mg GAE/g FW^54^. Lee et al.. revealed that the TPC of six blackberry cultivars varied between 13.45 and 22.49 mg gallic acid equivalent/g DW^55^. TPC in three commercial blackberries ranged from 12.1 to 23.5 mg gallic acid equivalent/g DW^56,57^. In the same context, the total flavonoid content reported herein agreed with previous reports. Albert et al. reported the total flavonoid content in *R. fruticosus* extracts prepared with different extraction solvents ranged from 1.29 to 2.40 mg quercetin/g DW^58^. Besides, Celant et al. reported that the total flavonoid concentration varied between 0.46 ± 0.02 and 1.14 ± 0.01 mg quercetin/g FW^54^. The total phenolic and flavonoid contents in blackberries may differ depending on the variety, environmental conditions, harvest timing, and extraction method^58^.

### Characterization of *Rubus fruticosus* fruit extract (RFE) using UPLC-ESI-MS/MS

**Fig. 1:**
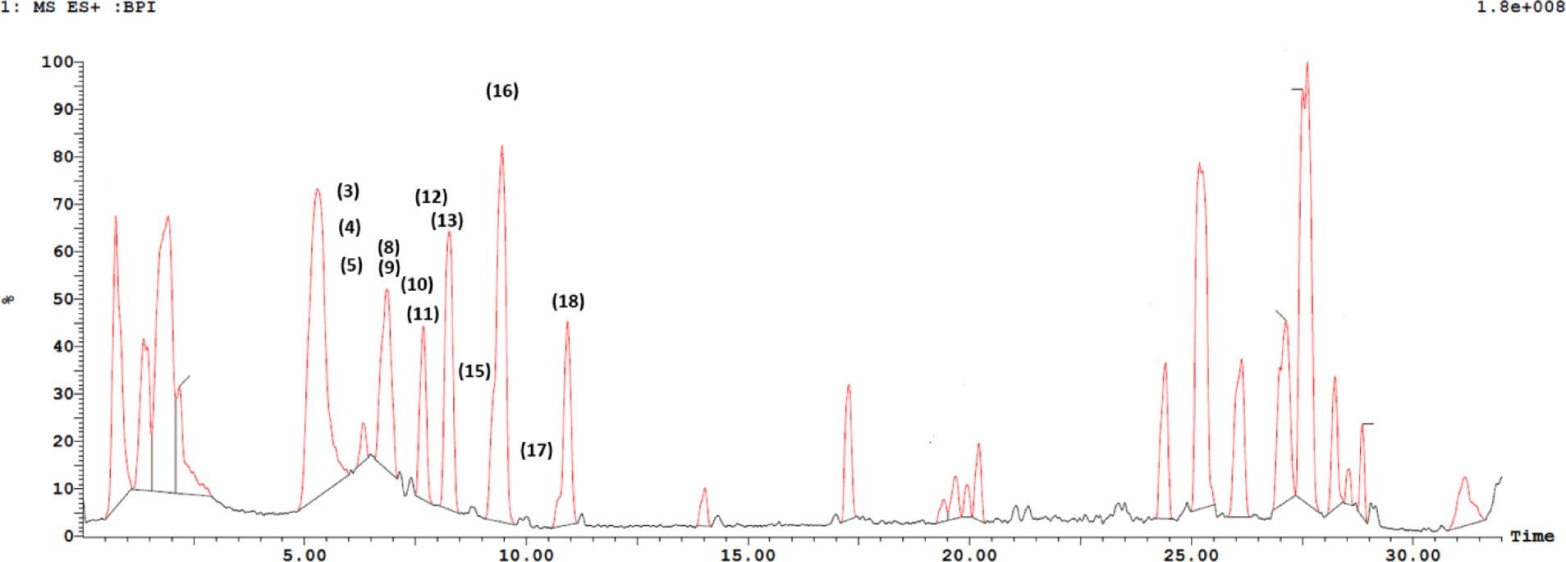
UPLC-ESI-MS base peak chromatogram of *Rubus fruticosus* L. fruit extract in the positive ion mode.

**Fig. 2:**
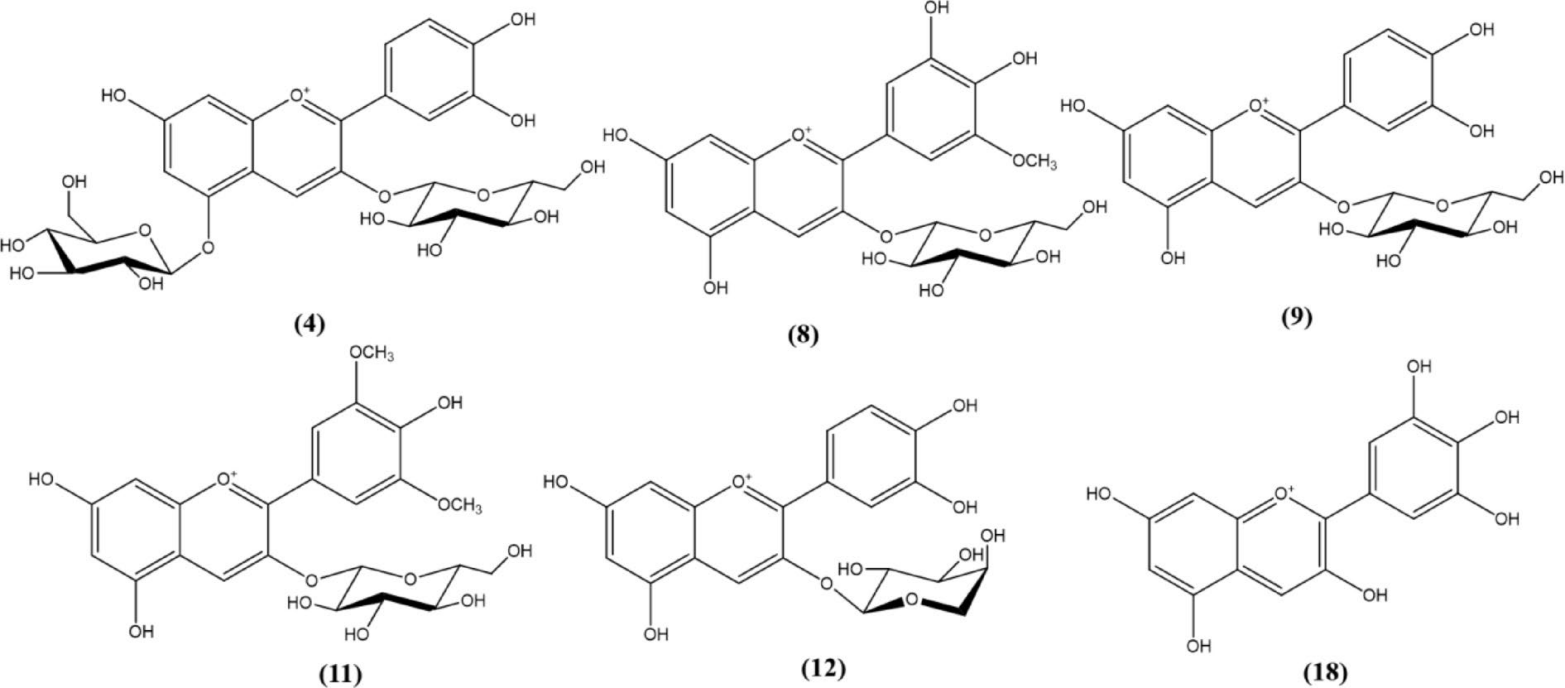
Major metabolites identified in *Rubus fruticosus* L. fruit extract.

Metabolites were assigned in *R. fruticosus* fruit extract using UPLC-ESI-MS/MS in both positive and negative ion modes (Table 2; Fig. 1). Peak assignments were carried out based on the molecular weight and structural information obtained from their MS spectra, in addition to retention times of the eluted compounds, with those reported for *R. fruticosus* and online public databases (Table 2). Nine anthocyanins, three anthocyanidins, three flavonols, and one phenolic acid were identified in the fruit extract (Table 2). Anthocyanin glycosides dominated *R. fruticosus* fruit extract, and five of the main identified glycosides were characterized as cyanidin derivatives due to a fragment ion at *m/z* 287 in the positive ionization mode, corresponding to cyanidin aglycone, which was further glycosylated with different sugars. Cyanidin glycosides including cyanidin-*O*-hexoside (peak **9**) with the respective parent ion (*m/z* 449), cyanidin-*O*-di and trihexosides with their characteristic parent ions (*m/z* 611 and 773, respectively), cyanidin-*O*-rutinoside (peak **10**) with a parent ion at *m/z* 595 and cyanidin-*O*-pentoside (peak **12**) exhibiting a pseudomolecular ion at *m/z* 419 were identified in the investigated extract^59–61^. Malvidin-*O*-hexoside (peak **11**) was also identified (*m/z* 493) with a fragment ion at *m/z* 331 corresponding to malvidin aglycone and was in agreement with the previous report by Cho et al.^62^. Three flavonol glycosides were annotated displaying pseudomolecular ions at *m/z* 447, 433 and 609, respectively, which were identified as kaempferol-*O*-hexoside (peak **6**), quercetin-*O*-pentoside (peak **7**) and quercetin-*O*-rutinoside (peak **14**), respectively, in accordance with previously reported data (Fig. 2)^63–67^.

**Table 2:** Metabolites assigned *Rubus fruticosus* L. fruit extract *via* UPLC-ESI-MS/MS in negative and positive ion modes.

Peak #	Rt (min)	Metabolite	[M-H]^−^	M^+^	MS^2^ ions (m/z)	References
1	0.87	Chlorogenic acid	353	–	nd	^36^
2	0.87	Quinic acid	191	–	nd	^68^
3	6.33	Cyanidin-*O*-trihexoside	–	773	287	
4	6.33	Cyanidin-*O*-dihexoside	–	611	287	^60^
5	6.33	Pelargonidin-*O*-rutinoside	–	579	257, 110, 109	^69^
6	6.51	Kaempferol-*O*-hexoside	447	–	285, 284	^60^
7	6.51	Quercetin-*O*-pentoside	433	–	81	^63^
8	6.94	Petunidin-*O*-hexoside	–	479	227, 128 (100)	^61^
9	6.94	Cyanidin-*O*-hexoside	–	449	287	^59^
10	7.00	Cyanidin-*O*-rutinoside	–	595	287	^60^
11	7.59	Malvidin-*O*-hexoside	–	493	331, 299 (100), 224, 173, 147	^62^
12	7.59	Cyanidin-*O*-pentoside	–	419	287, 257, 235, 127, 109 (100)	^61^
13	7.64	Delphinidin-*O*-acetyl hexoside	–	507	261, 187, 110	^61^
14	8.61	Quercetin-*O*-rutinoside (Rutin)	609		393	^66^
15	8.82	Petunidin	–	317	195, 166, 153, 139, 109 (100)	^61^
16	9.51	Malvidin	–	331	216, 196, 181, 155, 139, 125, 109 (100)	^61^
17	10.29	Petunidin-*O*-hexoside	–	479	nd	^62^
18	10.93	Delphinidin	–	303	nd	^61^
19	17.73	Quercetin	301	–	300 (100)	^68^

UPLC-ESI-MS/MS analysis of Rubus fruticosus L. fruit extract in both ion modes. The metabolites assigned for the extract are revealed with associated references.

### UV scanning of *Rubus fruticosus *fruit extract in 2% acetic acid

The spectrophotometric analysis of *Rubus fruticosus* extract showed absorption bands at 283 nm and 530 nm which went parallel with previous studies^70^. All subsequent measurements were done at wavelength 283 nm (λ _max_).

### Linear regression analysis of *Rubus fruticosus *fruit extract in 2% acetic acid

**Fig. 3:**
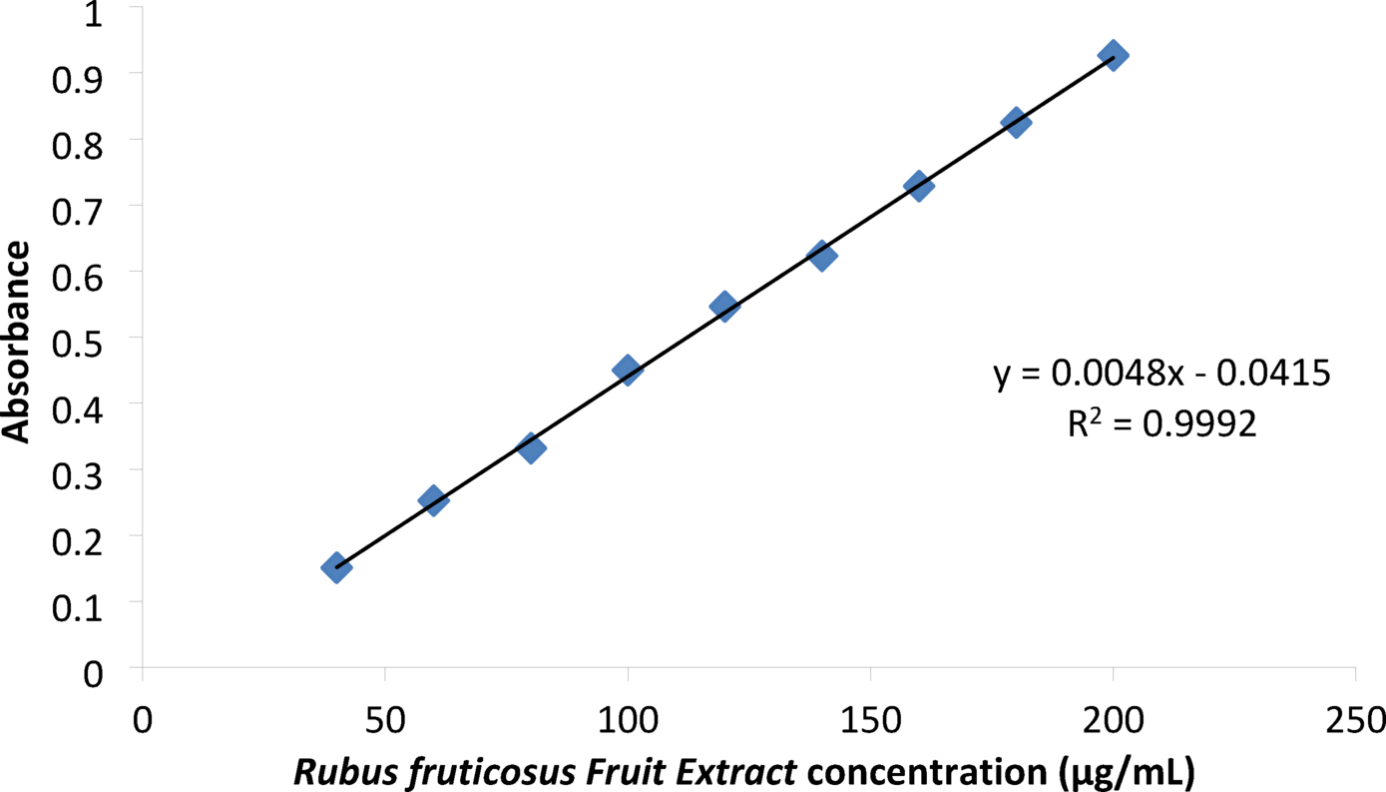
Calibration curve of *Rubus fruticosus Fruit Extract* in 2% acetic acid at λ_max_ 283 nm.

Table 3 and Fig. 3showed linear relationships between absorbances and corresponding concentrations of *R. fruticosus* fruit extract in 2% acetic acid, obeying Beer Lambert’s law. The figure shows the linear equation and coefficient of regression (R^2^.

**Table 3:** Relationship between various concentrations of *Rubus fruticosus fruit extract* and UV absorbances at 283 nm (λ_max_) in 2% acetic acid.

Rubus fruticosus extract concentration (µg/mL)	Absorbance
40	0.1509
60	0.2524
80	0.3318
100	0.4498
120	0.5462
140	0.623
160	0.7286
180	0.8247
200	0.9264

### Characterization of the prepared *Rubus fruticosus* fruit extract/Chitosan nanoparticles

Table 4 shows all the physicochemical properties of the prepared CSNPs. It is shown that P.S. increased significantly (*p* < 0.05) with increasing chitosan concentration from 0.1 to 0.3% and by increasing CS: TPP volume ratio from 3:1 to 6:1 v/v. Meanwhile, no significant changes were observed in PDI, ξ potential, or %EE.

The significant decrease in P.S. with the volume ratio from 6:1 to 3:1 was attributed to an increase in concentration of TPP, which causes more cross-linking within the formed NPs, causing shrinkage of the NPs. It is noteworthy that all PDI values are > 0.5, which indicates some sort of aggregation. Moreover, all formulae carried a positive charge due to NH3^+^ of chitosan, which is responsible for the mucoadhesive properties. F1 was chosen for further optimization.

It is noteworthy that by reducing the pH of TPP, a significant reduction in the P.S and PDI together with a significant increase in the EE% (*p* < 0.05) was observed. At lower pH values, TPP becomes less reactive for electrostatic interactions with chitosan because it is buffered by more positive ions in solution (H3O^+^ and H^+^). TPP therefore reacts with fewer amino groups of chitosan (NH3^+^), leading to the formation of smaller-sized nanoparticles that are more monodisperse^71^. Additionally, the highly protonated chitosan at acidic pH causes stronger repulsion between NPs (Supplementary Fig. 1).

Meanwhile, anthocyanins tend to be soluble in acidic conditions, which makes it easier to be encapsulated by CSNPs^72^. At a lower pH or in an acidic condition, anthocyanins are highly soluble in water due to the formation of flavylium cation which appears as red^73^. Meanwhile, the addition of the drug to the TPP solution (F7) didn’t show any significant (*p* > 0.05) changes compared to F6. F6 was chosen for the in vivo study. F6 was selected for the in vivo studies due to its optimal particle size (194.49 ± 5.69 nm) and low PDI (0.402 ± 0.035). Meanwhile, it showed the highest encapsulation efficiency (64.6 ± 1.12%).

**Table 4:** Characterization of the prepared *Rubus fruticosus*/chitosan nanoparticles (RF-CSNPs).

Formula code	*P*.S (nm)	PDI	ξ potential (mV)	EE (%)
F1	334.5 ± 4.82	0.639 ± 0.083	38.7 ± 0.65	52.58 ± 3.31
F2	721.4 ± 3.50	0.714 ± 0.017	39.5 ± 0.35	51.74 ± 4.12
F3	2273.6 ± 58.35	0.632 ± 0.092	41.2 ± 0.43	50.34 ± 2.35
F4	2968.7 ± 75.92	0.699 ± 0107	40.6 ± 0.76	51.27 ± 3.36
F5	247.32 ± 5.37	0.427 ± 0.023	39.5 ± 0.72	62.44 ± 0.57
F6	194.49 ± 5.69	0.402 ± 0.035	38.6 ± 0.45	64.6 ± 1.12
F7	196.35 ± 4.23	0.415 ± 0.015	39.2 ± 0.31	59.55 ± 0.64

F1–F4 were prepared without pH adjustment (TPP, pH 9).

F5–F7 were prepared after pH adjustment of F1, F5 (TPP, pH 7), F6, and F7 (TPP, pH 2).

In all preparations, the drug was added to the chitosan solution, except in the case of F7, where the drug was added to TPP solution.

### Behavioral tests

#### Locomotor activity

**Fig. 4:**
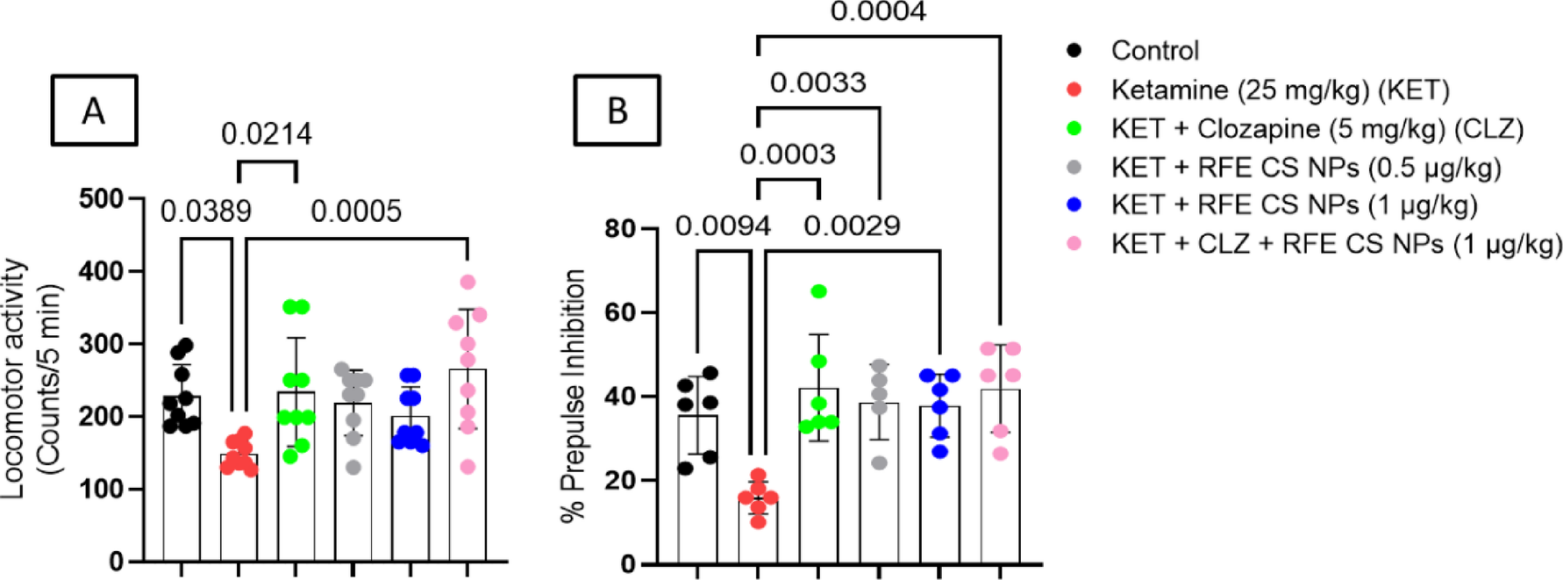
Effect of REF/CLZ treatment on locomotor activity (**A**) and %PPI (**B**) against ketamine-induced schizophrenia in rats. Data are presented as mean ± SD (*n* = 9 and 6) using one-way ANOVA followed by Tukey as a post-hoc test (*P* < 0.05).

The ketamine-treated group illustrated a significant decrease in locomotor activity by 34.79% compared to the control group (F_(5,48)_ = 4.56). On the other side, treatment with CLZ and a combination of CLZ/RFE (1 mg/kg) revealed a significant increase in locomotor activity as compared to the ketamine-treated group by 36.36% and 43.99%, respectively (Fig. 4A).

#### Acoustic startle response

Statistical analysis revealed significant differences among different groups on %PPI (F_(5,29)_ = 6.88). Ketamine-treated rats showed a significant reduction in %PPI compared to the control group by 2.24 folds. CLZ treatment showed a significant increase in %PPI as compared to ketamine-treated rats by 2.65 folds. Moreover, RFE (0.5 mg/kg), RFE (1 mg/kg), and a combination of CLZ and RFE (1 mg/kg) treatment illustrated a significant increase in %PPI as compared to ketamine-treated rats by 2.43, 2.38, and 2.63 folds, respectively (Fig. 4B).

#### Splash test

**Fig. 5:**
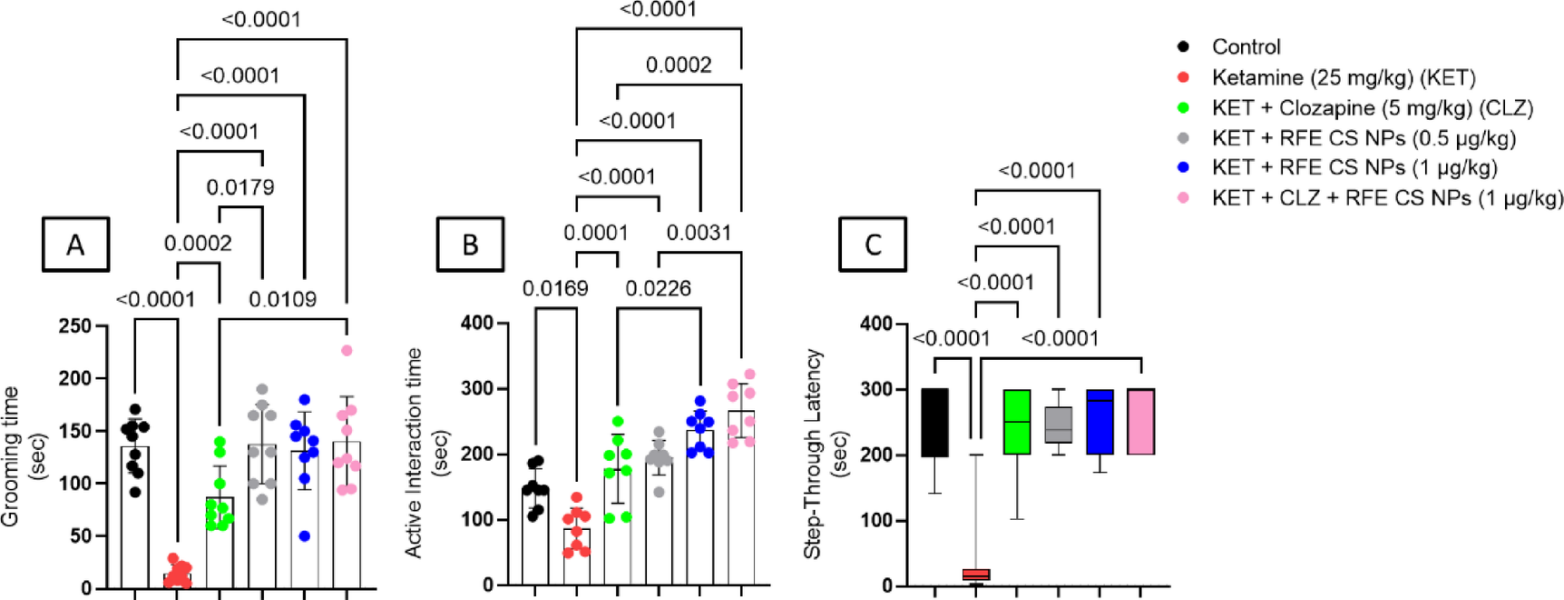
Effect of RFE/CLZ treatment on grooming time of splash test (**A**), social interaction (**B**), and passive avoidance (C) against ketamine-induced schizophrenia in rats. Data are presented as mean ± SD (*n* = 8) using one-way ANOVA followed by Tukey as a post-hoc test (*P* < 0.05).

Ketamine treatment reduces grooming time significantly by 9.2 folds relative to the control group, while treatment with CLZ, RFE (0.5 mg/kg), RFE (1 mg/kg), and a combination of CLZ and RFE (1 mg/kg) significantly enhanced grooming time by 5.89, 9.32, 8.88, and 9.49 folds, respectively, as compared to the ketamine-treated group. Moreover, treatment with both low doses of RFE showed a significant increase in grooming time as compared to the CLZ-treated group (Fig. 5A) (F_(5,48)_ = 21.5).

#### Social interaction

The active time of interaction was significantly decreased in ketamine-treated groups when compared to control, CLZ-treated, RFE-treated (0.5 mg/kg), RFE-treated (1 mg/kg), and combination-treated groups (F_(5,42)_ = 25.2). Interestingly, rats treated with either RFE (1 mg/kg) or a combination of CLZ/RFE illustrated significant elevation relative to CLZ-treated rats. Moreover, combination-treated and RFE-treated (1 mg/kg) rats showed significant elevation in active interaction time compared to RFE (0.5 mg/kg)-treated rats. Also, the combination-treated group showed a significant increase in active interaction time relative to RFE (0.5 mg/kg)-treated rats (Fig. 5B).

#### Step-through passive avoidance

Kruskal–Wallis test revealed that ketamine treatment resulted in a significantly shorter latency to step through compared to the control group, while treatment with CLZ, RFE (0.5 mg/kg), RFE (1 mg/kg), and a combination of CLZ and RFE (1 mg/kg) significantly attenuated ketamine-induced step-through (Fig. 5C) (F_(5,48)_ = 22.3).

### Effect of RF-CSNPs/CLZ on antioxidant enzymes in ketamine-induced schizophrenia in rats

#### Catalase

**Fig. 6:**
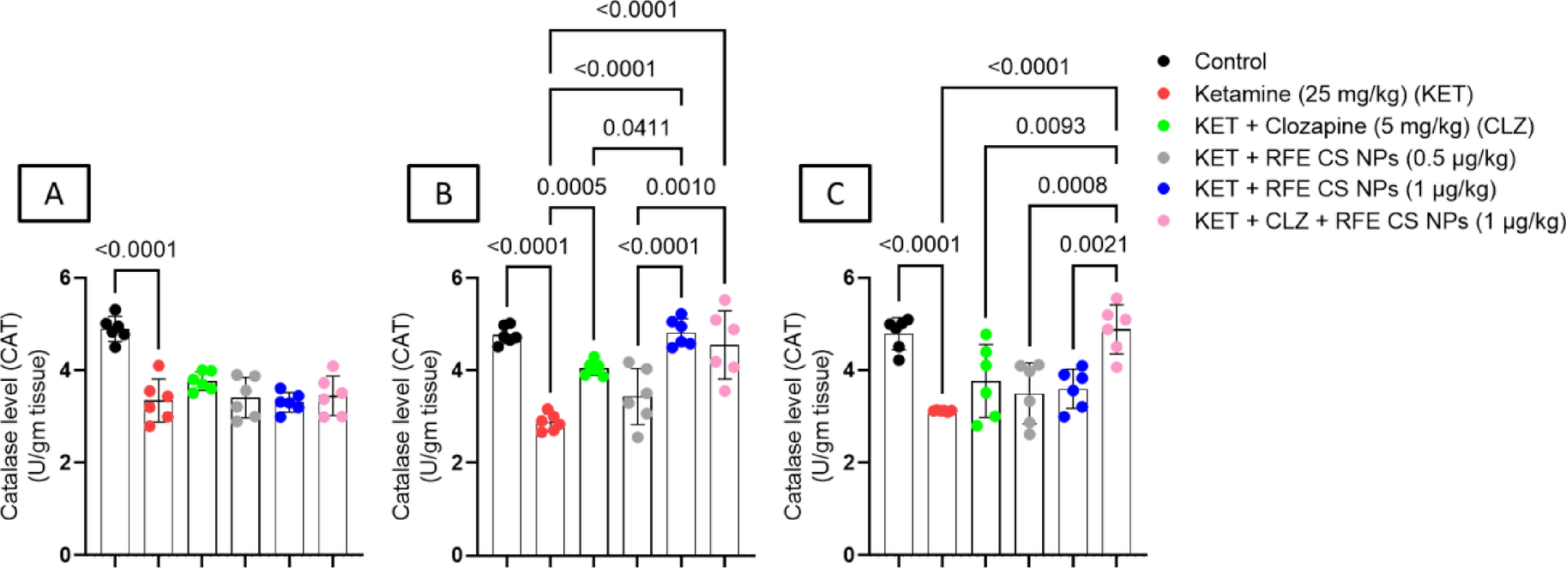
Effects of ketamine and REF/CLZ on striatal, prefrontal cortical, and hippocampal catalase levels in schizophrenia-induced rats. Ketamine was administered for 14 days. RFE and CLZ were administered for 7 days, from day 8 to day 14. Data are presented as means ± S.D. (*n* = 6). The significance level was *P* < 0.05 using a one-way analysis of variance (ANOVA) followed by Tukey as a post-hoc test.

Ketamine-treated rats showed a significant decrease in catalase levels as compared to the corresponding control group in the striatum (Fig. 6A) (F_(5,30)_ = 18), prefrontal cortex (Fig. 6B) (F_(5,30)_ = 20.4), and hippocampus (Fig. 6C) (F_(5,30)_ = 11.5). In the prefrontal cortex, treatment with either CLZ, RFE (1 mg/kg), or a combination of CLZ and RFE (1 mg/kg) revealed a significant increase in catalase level as compared to ketamine-treated rats. Interestingly, RFE (1 mg/kg)-treated and combination-treated rats illustrated a significant increase in prefrontal cortical catalase levels as compared to RFE-treated (0.5 mg/kg) rats. Also, CLZ treatment exhibited a significant reduction in catalase level relative to the RFE-treated (1 mg/kg) group. Concerning the hippocampus, the combination-treated group revealed a significant increase in catalase level as compared to ketamine-treated, CLZ-treated, RFE-treated (0.5 mg/kg), and RFE-treated (1 mg/kg) rats (Fig. 6).

#### Reduced glutathione

**Fig. 7:**
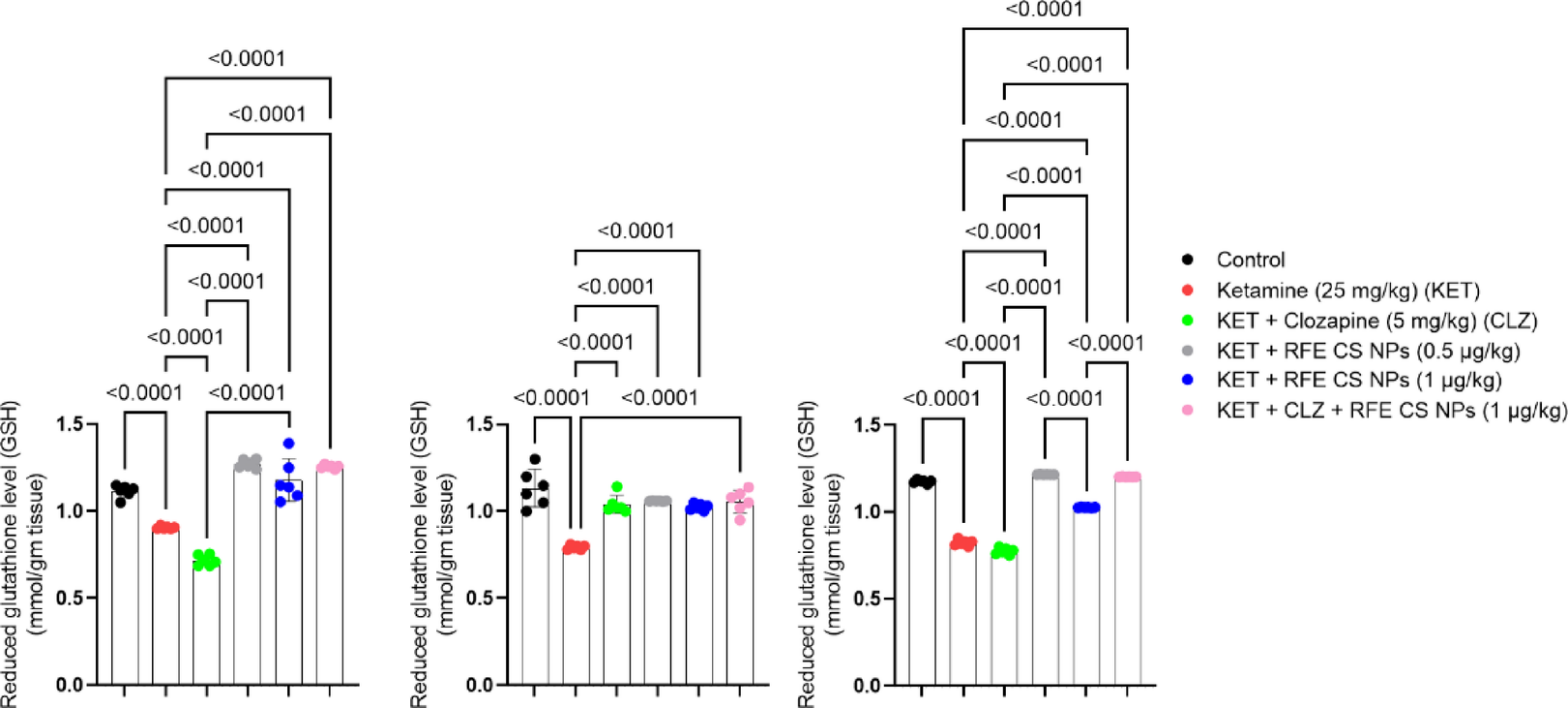
Effects of ketamine and REF/CLZ on striatal, prefrontal cortical, and hippocampal reduced glutathione level in schizophrenia-induced rats. Ketamine was administered for 14 days. RFE and CLZ were administered for 7 days, from day 8 to day 14. Data are presented as means ± S.D. (*n* = 6). The significance level was *P* < 0.05 using one-way analysis of variance (ANOVA) followed by Tukey as a post-hoc test.

Ketamine-treated rats showed a significant decrease in reduced glutathione levels as compared to the corresponding control group in the striatum (Fig. 7A) (F_(5,30)_ = 95.8), prefrontal cortex (Fig. 7B) (F_(5,30)_ = 25.2), and hippocampus (Fig. 7C) (F_(5,30)_ = 1818). On the other side, treatment with RFE (0.5 mg/kg), RFE (1 mg/kg), and a combination of CLZ and RFE (1 mg/kg) illustrated a significant increase in striatal reduced glutathione levels as compared to both ketamine-treated and CLZ-treated rats. Concerning the prefrontal cortex, treatment with CLZ, RFE (0.5 mg/kg), RFE (1 mg/kg), and a combination of CLZ and RFE (1 mg/kg) revealed a significant elevation in GSH level relative to a ketamine-treated group of rats. Regarding hippocampus tissue, RFE (0.5 mg/kg), RFE (1 mg/kg), and a combination of CLZ and RFE (1 mg/kg) showed a marked increase in GSH levels compared to ketamine-treated and CLZ-treated rats. Interestingly, RFE (0.5 mg/kg)-treated and combination-treated rats showed a significant increase in hippocampal reduced glutathione as compared to RFE-treated (1 mg/kg) rats (Fig. 7).

### **Effect of RF-CSNPs/CLZ on*TNF-α*level in ketamine-induced schizophrenia in rats**

**Fig. 8:**
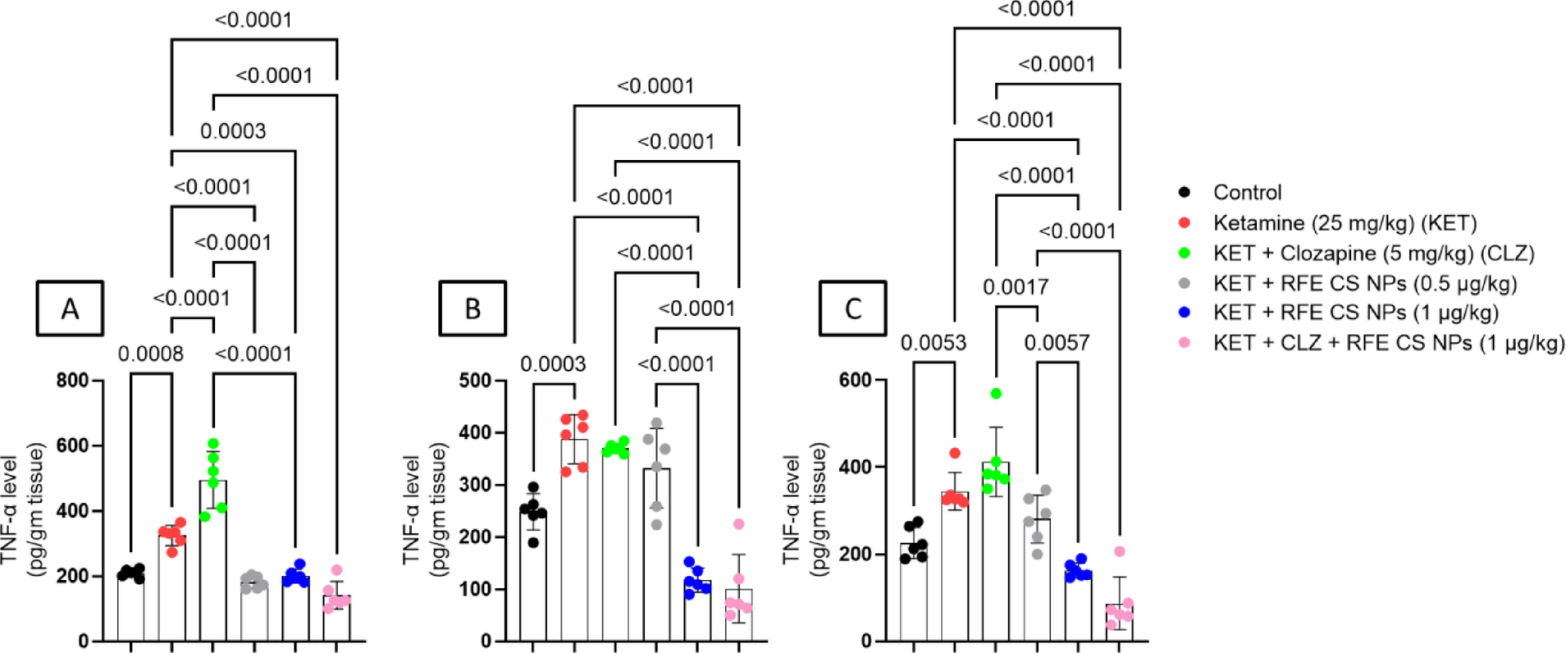
Effects of ketamine and REF/CLZ on striatal, prefrontal cortical, and hippocampal TNF-α levels in schizophrenia-induced rats. Ketamine was administered for 14 days. RFE and CLZ were administered for 7 days, from day 8 to day 14. Data are presented as means ± S.D. (*n* = 6). The significance level was *P* < 0.05 using one-way analysis of variance (ANOVA) followed by Tukey as a post-hoc test.

Ketamine treatment showed a significant increase in TNF-α levels relative to the corresponding control group in all studied brain areas. Concerning striatum (Fig. 8A) (F_(5,30)_ = 54.6), RFE (0.5 mg/kg), RFE (1 mg/kg), and a combination of CLZ treatment showed a significant decrease in TNF-α as compared to ketamine-treated and CLZ-treated rats. In the prefrontal cortex (Fig. 8B) (F_(5,30)_ = 40.6), high-dose anthocyanin and combination treatment illustrated a significant decrease in TNF-*α* levels as compared to ketamine-treated, CLZ-treated, and low-dose RFE-treated groups. For the hippocampus (Fig. 8C) (F_(5,30)_ = 30.9), RFE-treated (1 mg/kg) and combination-treated groups demonstrated a significant decrease in TNF-*α* levels relative to ketamine-treated, CLZ-treated, and RFE-treated (0.5 mg/kg) groups (Fig. 8).

### **Effect of RF-CSNPs/CLZ on BDNF level in ketamine-induced schizophrenia in rats**

**Fig. 9:**
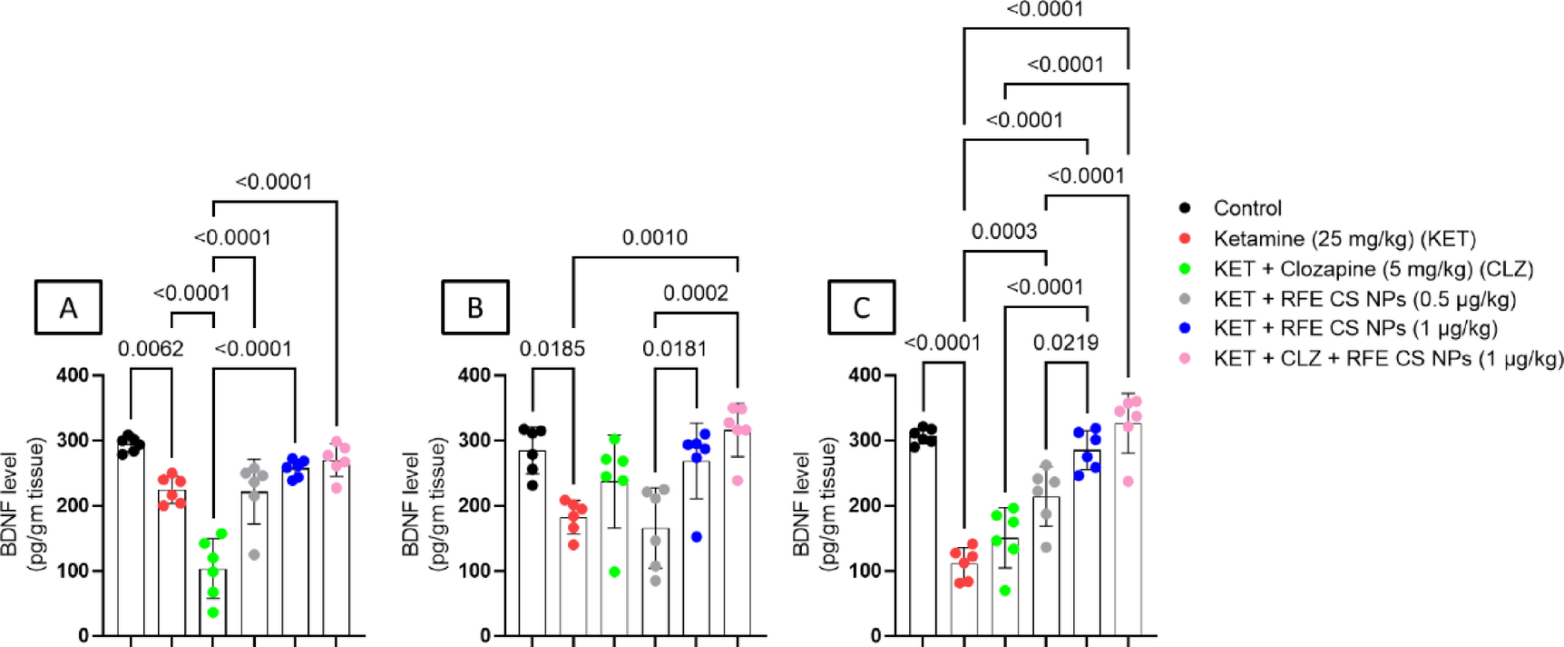
Effects of ketamine and REF/CLZ on striatal, prefrontal cortical, and hippocampal BDNF in schizophrenia-induced rats. Ketamine was administered for 14 days. RFE and CLZ were administered for 7 days, from day 8 to day 14. Data are presented as means ± S.D. (*n* = 6). Significance level was *P* < 0.05 using one way analysis of variance (ANOVA) followed by Tukey as a post-hoc test.

Ketamine treatment showed a significant decrease in BDNF levels relative to the corresponding control group in all brain areas studied. Concerning the striatum (Fig. 9A) (F_(5,30)_ = 27.4), RFE (0.5 mg/kg), RFE (1 mg/kg), and a combination of CLZ/RFE (1 mg/kg) showed a significant increase in BDNF as compared to CLZ-treated rats. In the prefrontal cortex (Fig. 9B) (F_(5,30)_ = 35.2), combination-treated groups illustrated significant elevation in BDNF level as compared to the ketamine-treated group. Interestingly, RFE (1 mg/kg) and a combination of CLZ RFE (1 mg/kg) revealed a significant elevation in BDNF level relative to RFE (0.5 mg/kg)-treated rats. For the hippocampus (Fig. 9C) (F_(5,30)_ = 7.88), RFE (0.5 mg/kg), RFE (1 mg/kg), and a combination of CLZ/RFE (1 mg/kg) showed a significant increase in BDNF as compared to ketamine-treated rats. Interestingly, RFE (1 mg/kg) and a combination of CLZ/RFE (1 mg/kg) revealed a significant elevation in BDNF level relative to CLZ-treated and RFE (0.5 mg/kg)-treated rats (Fig. 9).

### Effect of RF-CSNPs/CLZ on CLZ side effects in ketamine-induced schizophrenia in rats

#### Change in body weight

**Fig. 10:**
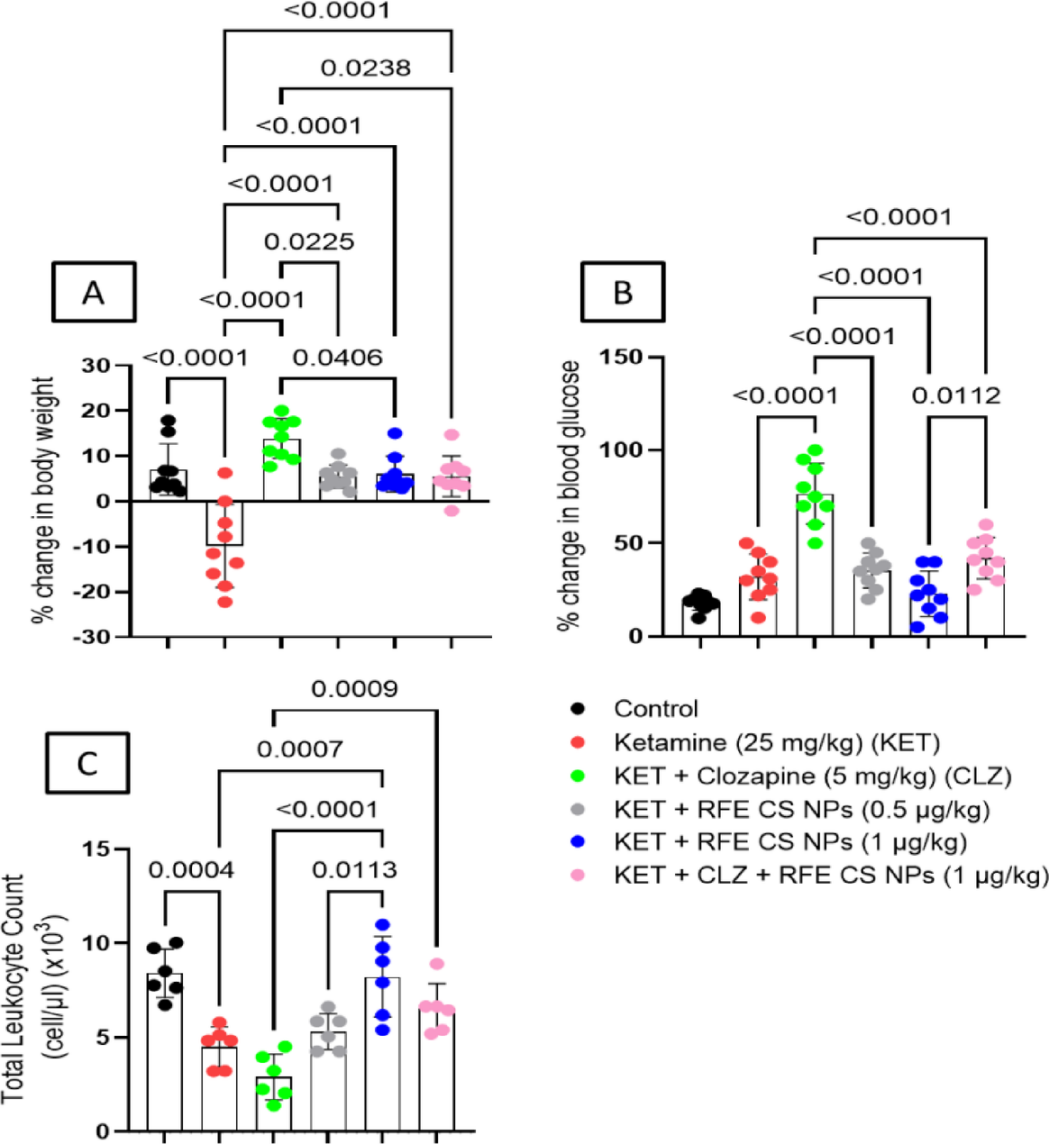
Effect of RFE/CLZ treatment on ketamine-treated rats on % change in body weight (**A**), % change in blood glucose (**B**), and Total leukocyte count. (**C**), at *p* < 0.05 (*n* = 6) using one-way ANOVA followed by Tukey as a post-hoc test.

Ketamine-treated rats presented a significant reduction in % change in body weight relative to the control group (F_(5,48)_ = 18.4). However, the CLZ-treated group showed a significant increase in % change in body weight compared to all other groups. On the other hand, ketamine treatment significantly reduced the % change in body weight compared to other treated groups (Fig. 10A).

#### Blood glucose

CLZ treatment showed a significant increase in % change in blood glucose relative to other groups (F_(5,48)_ = 29.5). However, the RFE (1 mg/kg)-treated group illustrated a significant decrease in % change in blood glucose relative to the combination-treated group (Fig. 10B).

#### Total leukocyte count

The group of rats treated with CLZ illustrated a significant decrease in total leukocyte count compared to the RFE (1 mg/kg) treatment or the combination-treated group (F_(5,48)_ = 14.6). In addition, the ketamine-treated group illustrated a significant reduction in total leukocyte count compared to control and RFE (1 mg/kg)-treated groups. Moreover, RFE (1 mg/kg) treatment showed significant elevation in total leukocyte count relative to the RFE (0.5 mg/kg)-treated group (Fig. 10C).

#### Serum total cholesterol and triglycerides

**Fig. 11:**
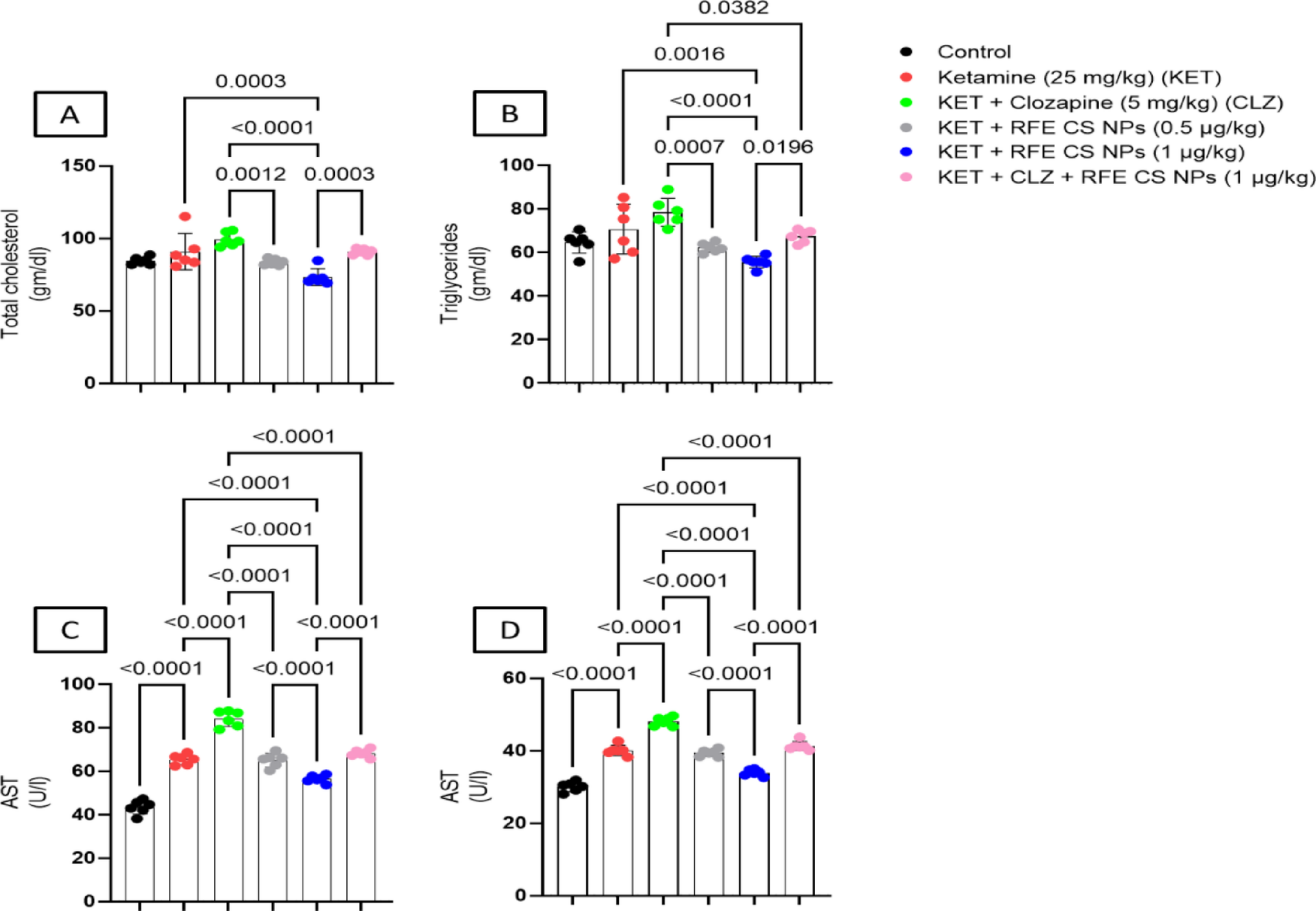
Effect of RFE/CLZ treatment on ketamine-treated rats on total cholesterol (**A**), serum triglycerides (**B**), AST (**C**), and ALT (**D**). Data are presented as mean ± SD (*n* = 6), Statistical significance was detected at *p* < 0.05, using one-way ANOVA followed by Tukey as a post-hoc test.

For total cholesterol level, CLZ treatment enhanced significantly the total cholesterol level as compared to both anthocyanin-treated groups (F_(5,30)_ = 12.2). In addition, RFE (1 mg/kg) treatment significantly reduced serum cholesterol relative to the ketamine-treated group by 1.24 folds (Fig. 11A). For serum triglycerides, ketamine treatment revealed increased triglycerides level as compared to RFE (1 mg/kg)-treated group, while CLZ-treated rats revealed significantly higher triglycerides level relative to rats treated with RFE (0.5 mg/kg), RFE (1 mg/kg), and combination of CLZ/RFE (Fig. 11B) (F_(5,48)_ = 10.0).

#### Serum AST and ALT

Ketamine treatment significantly elevated ALT and AST levels, indicating hepatic damage, which was mitigated by RFE treatment, suggesting hepatoprotective effects. Ketamine treatment showed significant enhancement for ALT and AST as compared to the corresponding control group. On the other hand, treatment with RFE (1 mg/kg) revealed a significant decrease in AST level compared to the ketamine-treated group (F_(5,30)_ = 146). Surprisingly, CLZ-treated rats showed a significant increase in AST level as compared to RFE (0.5 mg/kg), RFE (1 mg/kg), and combination-treated groups. In addition, RFE (1 mg/kg) treatment showed a significantly reduced serum AST level relative to RFE-treated (0.5 mg/kg) and combination-treated rats (Fig. 11C), Regarding ALT level (Fig. 11D), CLZ treatment showed marked elevation as compared to all other treatment groups (F_(5,30)_ = 161). Moreover, RFE (1 mg/kg) treatment showed a significantly reduced serum AST level relative to RFE-treated (0.5 mg/kg) and combination-treated rats.

## Discussion

Repeated ketamine administration in rats has been shown to induce a spectrum of schizophrenia-like symptoms, including positive, negative, and cognitive manifestations^74^. This effect is partially attributed to ketamine’s activity as an NMDA receptor antagonist. Specifically, NMDA receptor blockade within limbic and subcortical brain regions can lead to elevated glutamate, dopamine, and serotonin release, which may contribute to the onset of positive symptoms^75^. Additionally, ketamine’s impact on NMDA receptors in the ventral tegmental area has been linked to reduced dopamine, serotonin, and GABA release in the prefrontal cortex, potentially eliciting negative symptoms^76^. Furthermore, ketamine treatment has been reported to increase the levels of reactive oxygen species, including superoxide anions, hydrogen peroxide, and hydroxyl radicals, while decreasing catalase activity, thereby promoting oxidative stress^77^. Based on these effects, ketamine was employed in this study as a preclinical model for schizophrenia.

In the present study, positive, negative, and cognitive symptoms were assessed using behavioral tests. Firstly, locomotor activity and PPI tests were used to assess positive symptoms. Ketamine treatment reduced both locomotor activity and startle response^42^. On the other hand, treatment with CLZ, anthocyanin-rich extract of *R. fruticosus* (RFE) in both doses, and a combination of RFE and CLZ improved startle response. Surprisingly, the RFE/CLZ combination showed superior results to other treated groups in locomotor activity. In addition, treatment with *R. fruticosus* fruit extract at 0.5 mg/kg and in combination with CLZ showed superior results to CLZ treatment concerning the sucrose splash test. The high dose of the fruit extract either alone or in combination with CLZ illustrated enhanced social interaction relative to the CLZ-treated group. Thus, the *R. fruticosus* extract showed superior effects on the negative symptoms. Concerning cognitive symptoms, RFE (both doses), CLZ treatment, and the combination of RFE/CLE treatment illustrated significant improvement in short-term memory compared to the ketamine-treated group as evidenced by step-through passive avoidance test results.

In vitro studies have revealed that a blackberry-supplemented diet effectively attenuated motor and cognitive performance, especially working memory, and showed increased hippocampal neurogenesis and expression of insulin-like growth factor 1 in aged rats^78,79^. Additionally, blackberry extract reversed the impaired cognitive functions related to memory and learning in diabetic rats^16^. Moreover, berry-based supplements or foods have successfully improved rain perfusion, cognitive function, memory performance, executive functioning, processing speed, and attention indices in older adults^80^. The observed changes of reversing age-related declines in brain function and cognitive and motor performance in rats were mainly related to the presence of polyphenolic compounds, especially anthocyanins, increasing antioxidant and/or anti-inflammatory levels, or by direct effects on neurogenesis and signaling in the brain involved in learning and memory^78,81^.

Blackberries are recognized for their high phenolic content, including flavonoids, phenolic acids, tannins, and anthocyanins^14,15^. Anthocyanins, the main class in *R. fruticosus* extract, are a group of naturally occurring phenolic compounds related to the coloring of plants, flowers, and fruits. These pigments are important as quality indicators and as chemotaxonomic markers^82,83^. Anthocyanins are absorbed as glycosides in rodents and humans^84,85^. Dietary supplementation with fruit or vegetable extracts showed that anthocyanins demonstrated the highest efficacy in penetrating the brain cell membrane and in providing antioxidant protection^81^.

Anthocyanins exhibited a neuroprotective effect on acetylcholinesterase activity, attenuated scopolamine-induced amnesia, and restored the Na^(+)^, K^(+)^-ATPase, and Ca^(2+)−^ATPase activities in rats with cognitive deficits associated with Alzheimer’s disease^86^. Additionally, shreds of evidence show that anthocyanins, with their different naturally occurring classes: Cyanidin, malvidin, delphinidin, petunidin, pelargonidin, and peonidin, may modulate neuronal cell death signaling pathways^87^ inhibit protein aggregation and potentiate autophagy^88,89^, along with maintaining calcium homeostasis that protects against excitotoxicity-induced neuronal cell death^90^. Moreover, anthocyanins and one of their major components, cyanidin-3-*O*-glucoside exhibited neuroprotective effects in various brain disorders, such as cerebral ischemia^91^. Alzheimer’s disease (AD)^92^, and Parkinson’s disease (PD)^93^.

Flavonoids are another important class of polyphenolic compounds present in *R. fruticosus* extract in the form of flavonol (quercetin and kaempferol) glycosides. Generally, flavonoids are divided into six classes based on their chemical skeleton: flavanols, flavanones, flavones, flavonols, isoflavonoids, and anthocyanidins^94^. While targeting multiple targets, they have been proven effective in preventing neurodegenerative disorders and in delaying the process of neurodegeneration^95^. Studies also suggested that flavonoids can cross the blood-brain barrier (BBB), which makes them potential agents in preventing neurodegenerative disorders^96,97^. Additionally, many studies have shown that quercetin and kaempferol glycosides exhibited neuroprotective effects in diseases like AD and PD, and scopolamine-induced memory impairment with improving learning, memory, and cognitive functions^98–102^.

Collectively, these effects may give some sort of evidence that an anthocyanin-rich extract of *R. fruticosus* could enhance negative symptoms more than CLZ and show the same results as CLZ in both positive and negative symptoms.

Ketamine-induced oxidative stress was evidenced by its effect on CAT and GSH levels in the striatum, prefrontal cortex, and hippocampus. RFE (1 mg/kg), both alone and in combination with CLZ, significantly enhanced prefrontal cortical CAT levels, highlighting its potential to counteract oxidative stress in schizophrenia. In addition, hippocampal CAT was enhanced with RFE (1 mg/kg) treatment, and the results were superior to CLZ and low-dose RFE treatment. Tena et al. reported that anthocyanins could act as hydrogen atom donors where free radicals are converted to more stable products^103^. In addition, anthocyanins could promote single-electron transfer, donating electrons to free radicals and reducing their oxidized intermediates into a more stable form. In addition, a significant elevation in striatal and hippocampal GSH was observed with RFE treatment at both doses and in combination with CLZ. Interestingly, RFE (0.5 mg/kg) and the combination of RFE/CLZ showed superior results in the prefrontal cortex, and the RFE/CLZ combination was superior in the striatum^104^. Experiments have shown that berry anthocyanins could penetrate a variety of cell types and that their incorporation into the cell’s membrane and cytosol protects against various oxidative stressors and loss of cell function^105^. Additionally, other studies have proved that one of the key mechanisms for the neuroprotective effects of anthocyanins is the suppression of neuroinflammation and oxidative stress, through which both anthocyanins and cyanidin-3-*O*-glucoside have been shown to protect neurons against cellular toxicities induced by factors like ischemia/reperfusion, pro-inflammatory cytokines^106^, 6-hydroxydopamine (6-OHPA)^93^, and hydrogen peroxide (H2O2)^107^. It also reversed nitric oxide/malonaldehyde (MDA) increase and superoxide dismutase (SOD) decrease in brain tissues in patients with cerebral ischemia^108^. Similarly, quercetin and kaempferol-3-*O*-*β*-D-glucuronate exhibited free radical scavenging activity against oxidative stress-mediated neuronal damage by modulating the expression of Nrf2-dependent antioxidant reactive elements and attenuating neuroinflammation^109–113^. Enhanced ROS production could induce an inflammatory response and promote the release of inflammatory cytokines, including TNF-α^114^.

Ketamine treatment enhanced TNF-α levels in all studied areas. Interestingly, RFE (1 mg/kg) and the combination of RFE/CFZ treatment showed superior results in the hippocampus and prefrontal cortex compared to other treated groups. Moreover, both doses of RFE and the combination of RFE/CLZ showed nearly the same results regarding the TNF-α level in the striatum, suggesting the protective effect of RFE on neuroinflammation. It was reported that RFE anti-inflammatory activity may be due to inhibition of NF-κB activation together with its underlying cytokines^115^. Also, bilberry anthocyanins improved memory and cognitive functions by reducing LPS brain levels and downregulating inflammatory mediators in the hippocampus, namely NF-ĸB, COX-2, iNOS, TNF-α, IL-1β, IL-6, and CD33^116^. Likewise, other findings related to improved motor disabilities in LPS-treated mice with the ability of anthocyanins to suppress the acute inflammatory response through the inhibition of JNK, ERK, p38, NF-κB, PI3 K/Akt, and MAPKs signaling pathways, preventing the upregulation of iNOS and COX-2 and the overproduction of TNF-α, IL-1β, prostaglandin E2 (PGE2) and IL-6 in mice brain^117–119^. Another study confirmed the ability of cyanidin-3-O-glucoside (C3G), the most common anthocyanin subfamily *in R. fruticosus*, to inhibit NF-κB and p38 MAPK pathways, suppressing the production of pro-inflammatory mediators, such as NO, PGE2, IL-1β, and IL-6^120^. Additionally, kaempferol glycosides exhibited neuroprotective effects against brain Injury and neuroinflammation by inhibiting the activation of NF-κB & STAT3 and upregulating endothelial nitric oxide synthase (eNOS) activity in transient focal stroke^121,122^. Kaempferol and quercetin flavonoids/glycosides also induced a down-regulation of the pro-inflammatory factors such as nitric oxide synthase, cyclooxygenase-2, and interleukin-1β and increased IκB-α, indicating a neuroprotective effect through attenuation of the inflammation^109^.

As a pro-inflammatory cytokine, TNF-α plays an important role in initiating and maintaining inflammation. Also, it was found that there is a mutual relationship between TNF-α and oxidative stress^123^. This crosstalk starts with oxidative stress, initiating the production of TNF-α, leading to cellular damage. On the other side, TNF-α can induce oxidative stress leading to apoptosis^124^.

Besides, its effect on oxidative stress, ketamine treatment reduced BDNF levels in the studied brain areas. However, both RFE-treated and RFE/CLZ-treated groups restored BDNF levels and showed superior results to the CLZ-treated group in the striatum. RFE (1 mg/kg) treatment, alone or in combination with CLZ, showed better results concerning prefrontal cortical and hippocampal BDNF. This could be explained partially by the anthocyanins’ effect on the Erk/CREB pathway^125^. Moreover, the dietary intake of flavonoids also improved human cognitive performance, mainly by exhibiting changes in serum BDNF and the related signaling apparatus^126^.

An interplay has been reported between the triad: oxidative stress, TNF-α, and BDNF. TNF-α was found to reduce the level of BDNF in neuronal cells, leading to neurodegeneration and reduction of synaptic plasticity. Also, enhanced oxidative stress has been found to reduce the level of BDNF. Elevated TNF-α can create a closed circuit where augmented inflammation leads to more oxidative stress, which in turn reduces BDNF levels, perpetuating neuronal injury. On the other hand, decreased BDNF can lead to reduced neuronal capacity to manage oxidative stress, further exacerbating the cycle of inflammation and damage^127^.

CLZ was previously reported for its side effects, which limit its use to resistant schizophrenia. CLZ may promote weight gain during treatment. This could be explained partly by enhancing lipid deposition and accumulation^128^. In our study, CLZ treatment increased body weight significantly. Interestingly, RFE, either alone or in combination with CLZ, diminished this effect and succeeded in returning body weight to normal. Many studies reported the positive influence of the consumption of anthocyanins and flavonoid-rich diets on preventing obesity and managing body weight and its associated metabolic syndrome (i.e., lipid profile) in both healthy and obese adults^115,129–132^. The anti-obesity activity of anthocyanins was mainly attributed to the suppression of the respective genes involved in lipogenesis and adipogenesis via downregulating the expression of NF-kB, PI3K, vascular endothelial growth factor 2 (VEGRF 2), *β*-catenin, Akt1, PPARγ, CCAAT, C/EBP*α*, aP2 (fatty acid-binding protein), FAS, and lipoprotein lipase (LPL)^133^. Additionally, other reports suggested that cyanidin and cyanidin-3-*O*-*β*-D-glucoside exhibited their anti-obesity potential by inhibiting pancreatic lipase enzyme or stimulating lipoprotein lipase (LPL) activity^134,135^.

CLZ treatment in the present study induced serum total cholesterol and triglycerides compared to anthocyanins-treated groups. Sterol regulatory element-binding protein (SREBP) may explain this CLZ-induced effect. CLZ-induced activation of SREBP promotes lipid and cholesterol production^136^. RFE treatment at both doses and in combination with CLZ reduced serum total cholesterol and triglycerides to nearly normal levels. This effect may be related to its inhibitory activity on SREBP, fatty acid and triglyceride synthesis, and lipogenic factors^137^. CLZ’s significant hyperglycemic effect may be related to its effect on serotonin and dopamine receptors, promoting hepatic glucose output and glucagon secretion^138,139^. These effects are in agreement with George et al.^140^. The elevation in serum cholesterol and triglycerides with CLZ treatment underscores the need for adjunct therapies like RFE to mitigate metabolic side effects in schizophrenia patients. CLZ was previously reported to induce hyperglycemia in a mouse model^141^. By contrast, RFE treatment reduced blood glucose levels at both doses. Anthocyanins were reported to ameliorate insulin resistance via enhancing PPAR-γ activity, and GLUT4 translocation, reducing SREBP expression and fatty acid synthesis^137^. In addition, other studies reported that blueberry supplementation reduced dyslipidemia (TC and LDL levels) and insulin resistance in DM patients and exhibited a significant level of weight and body fat reduction. The observed positive health benefits of blueberries were mainly attributed to its high content of anthocyanins and phenolic compounds^142,143^.

In the present study, CLZ induced leucopenia evidenced by low total leukocyte count. CLZ was reported to have inhibitory effects on neutrophil kinetics, leukocyte release from bone marrow, and neutrophil oxidative function^144^. RFE treatment, especially the high dose, corrected such elevation in total leukocyte count. This was reported previously by Fan et al.^145^. CLZ treatment may enhance liver toxicity, evidenced by increasing levels of liver transaminases via oxidative stress induction^146^. Both doses of RFE reduced AST and ALT levels relative to the CLZ treatment. Surprisingly, RFE/CLZ showed superior effects as compared to the CLZ-treated group. Anthocyanin’s inhibitory effect on oxidative stress and lipogenesis, in addition to its enhanced PPAR/lipolysis activity and up-regulating hepatic GSH levels, may explain its hepatoprotective activity^147–149^.

## Conclusion

The present study investigated the effects of blackberry-loaded chitosan nanoparticles on ketamine-induced schizophrenia in rats. Initially, an anthocyanin-rich blackberry extract (RFE) was prepared and characterized for its total phenolic and flavonoid content. Additionally, metabolites in RFE were identified using UPLC-ESI-MS/MS in both positive and negative ion modes. The encapsulation of RFE in chitosan nanoparticles significantly enhanced its therapeutic efficacy in addressing schizophrenia symptoms in rats. Administration of RFE-loaded vesicles via the intranasal route at doses of 0.5 and 1 mg/kg, or in combination with CLZ, produced effects comparable to, or superior to, standard CLZ treatment. This was confirmed by improvements in positive, negative, and cognitive symptoms of ketamine-induced schizophrenia, as assessed through behavioral testing. Furthermore, RFE mitigated the major side effects of clozapine, including its impact on body weight, blood glucose levels, total leukocyte count, serum cholesterol and triglycerides, and liver function. Through its antioxidant properties, RFE alleviated ketamine-induced oxidative stress by enhancing catalase activity and levels of reduced glutathione. RFE treatment also corrected the inflammatory response, as indicated by reduced levels of TNF-α. Additionally, BDNF levels were significantly elevated following RFE treatment. These findings suggest that RFE-loaded nanoparticles may offer a promising strategy to enhance the therapeutic potential of anthocyanins in the treatment of schizophrenia.

## Electronic supplementary material

Below is the link to the electronic supplementary material.

Supplementary Material 1

## Data Availability

The datasets generated during and/or analyzed during the current study are available from the corresponding author on reasonable request.
