# Supplementary figures and images for: Intranasal delivery of blackberry-loaded Chitosan nanoparticles for antipsychotic potential in Ketamine-induced schizophrenia in rats
Source: Sci Rep. 2025 May 14;15:16707. doi: 10.1038/s41598-025-00918-2 (PMC12078666; doi:10.1038/s41598-025-00918-2)

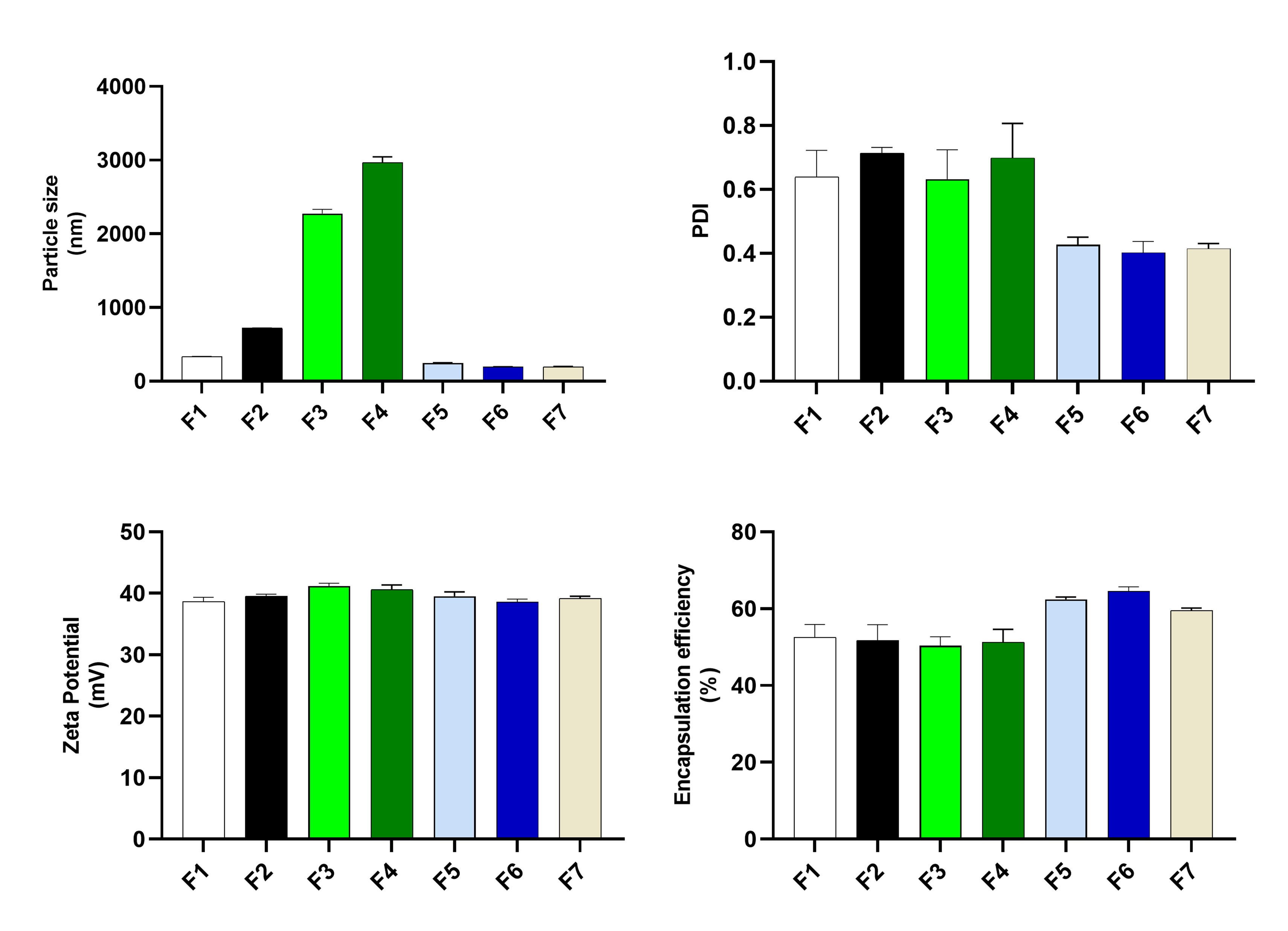

Supplement: Supplementary file 1 — Supplementary Material 1 [file 41598_2025_918_MOESM1_ESM.tif]
